# Large spin accumulation and crystallographic dependence of spin transport in single crystal gallium nitride nanowires

**DOI:** 10.1038/ncomms15722

**Published:** 2017-06-01

**Authors:** Tae-Eon Park, Youn Ho Park, Jong-Min Lee, Sung Wook Kim, Hee Gyum Park, Byoung-Chul Min, Hyung-jun Kim, Hyun Cheol Koo, Heon-Jin Choi, Suk Hee Han, Mark Johnson, Joonyeon Chang

**Affiliations:** 1 Center for Spintronics, Post-Si Semiconductor Institute, Korea Institute of Science and Technology, Hwarangno 14-gil 5, Seongbuk-gu, Seoul 02792, Korea; 2 Department of Materials Science and Engineering, Yonsei University, Seoul 03722, Korea; 3 Department of Nanomaterials Science and Engineering, Korea University of Science and Technology, Daejeon 34113, Korea; 4 KU-KIST Graduate School of Converging Science and Technology, Korea University, Seoul 02841, Korea; 5 Naval Research Laboratory, Washington, District Of Columbia 20375, USA

## Abstract

Semiconductor spintronics is an alternative to conventional electronics that offers devices with high performance, low power and multiple functionality. Although a large number of devices with mesoscopic dimensions have been successfully demonstrated at low temperatures for decades, room-temperature operation still needs to go further. Here we study spin injection in single-crystal gallium nitride nanowires and report robust spin accumulation at room temperature with enhanced spin injection polarization of 9%. A large Overhauser coupling between the electron spin accumulation and the lattice nuclei is observed. Finally, our single-crystal gallium nitride samples have a trigonal cross-section defined by the (001), (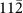) and (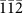) planes. Using the Hanle effect, we show that the spin accumulation is significantly different for injection across the (001) and (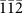) (or (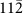)) planes. This provides a technique for increasing room temperature spin injection in mesoscopic systems.

## Abstract (web summary)

Semiconductors are promising for high performance spintronics but the low functioning temperature hampers their applications. Here the authors achieve a strong room temperature modulation of spin-dependent resistance in GaN nanowires, which marks an important step towards practical spintronic devices.

Recent basic research advances in spintronics have occurred at cryogenic temperature^1^,^2^,^3^ and include the realization of a spin-injected field-effect transistor and the observation of a gate-voltage-controlled conductance oscillation^4^,^5^. Numerous other devices with mesoscopic dimensions have demonstrated attractive properties for decades^6^,^7^,^8^,^9^,^10^. Recently, there have been a few meaningful achievements in spintronic devices at room temperature, such as a highly doped Si channel with tunnel contacts, a Heusler alloy/*n*-gallium arsenide spin valves, a spin logic device based on graphene and a gallium nitride (GaN)-based spin laser and spin valves^11^,^12^,^13^,^14^,^15^,^16^,^17^. However, the robust demonstration of spin transport in semiconductor devices at room temperature has remained elusive. Requirements for viable devices include a high spin-dependent resistance modulation, relatively low electrode contact and channel resistances, and a long spin diffusion length in a device with channel dimensions of tens of nanometres.

Here we demonstrate that single crystal GaN nanowires (NWs) show remarkably good room-temperature spin transport characteristics. Of greater importance, we observe a strong difference in spin accumulation for injection across the (001) and (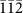) (or (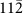)) planes.

## Results

### NW spin valve devices and measurement configurations

**Figure 1:**
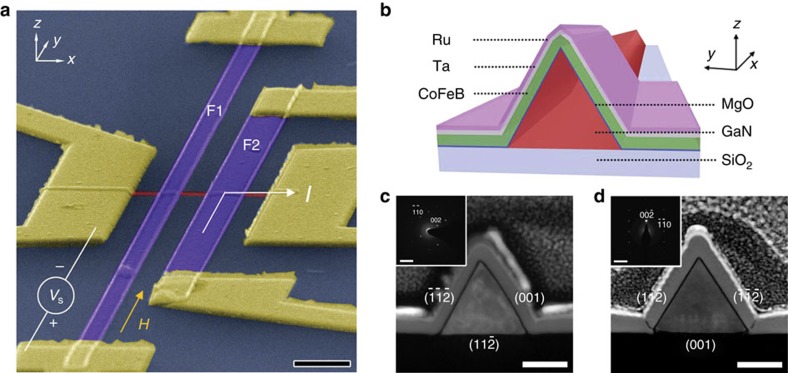
A lateral spin valve device with gallium nitride channel. (**a**) False-coloured scanning electron microscopy (SEM) image of a device (scale bar, 1 μm). The centre-to-centre channel length is *L*=1 μm. For measurements of MR dips, magnetic field *H* is applied along the easy axis (*y* axis) of spin detector F1 and spin injector F2. In studies of the Hanle effect, field *H* may be applied along *x*, *y* or *z*, or in the *y*–*z* plane at angle *θ* to *z*. (**b**) Schematic cross-sectional view of ferromagnetic electrode in contact with the GaN NW. (**c**,**d**) High-angle annular dark field images in scanning transmission electron microscopy mode showing views at the interface between the ferromagnetic tunnel electrode and GaN NW for two types of devices. Scale bars, 40 nm (**c**,**d**). The insets are the electron diffraction patterns with [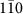] zone axis of the corresponding images with scale bars 5 nm^−1^. The selected area electron diffraction patterns show the surfaces of each GaN NW to have (001), (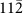) and (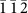) planes. (**c**) The (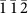) and (001) planes contact the ferromagnet in one device type (type-C device), whereas (**d**) the (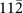) and (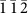) planes contact the ferromagnet in the second device type (type-D device).

The spin injection technique^18^,^19^,^20^ using the lateral nonlocal spin valve (NLSV) geometry is described in Fig. 1a, showing a GaN NW (red) and ferromagnetic thin-film electrodes. Biased with current *I*, a steady-state non-equilibrium population of polarized spins, spin accumulation 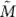, builds up near the F2/NW interface and diffuses along the NW. A voltage *V*_S_ ∝ 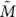, measured between spin detector F1 and an electrode at the far left end, depends on the relative magnetization orientations M1 and M2 of F1 and F2, respectively^4^,^18^. The ferromagnetic electrodes have different widths (300 and 600 nm) to ensure that portions of F1 and F2 in the *x*–*y* plane have different values of coercivity, *H*_C1_ ≠ *H*_C2_, so that ranges of field exist with M1 and M2 antiparallel when external field *H*_*y*_ is swept between −2.0 and 2.0 kOe.

Our GaN NWs are prepared by chemical vapour deposition and have lateral dimensions of about 80 nm with high uniformity along the entire length (Methods and Supplementary Note 1). Figure 1b shows a cross-section sketch of the NW and the ferromagnetic electrode formed as a tunnel junction, GaN/MgO/CoFeB/Ta/Ru. To investigate the structure of this junction in detail, cross-sectional samples were prepared by focused ion beam slicing and a lift-off process with a micromanipulator. Cross-sectional high-angle annular dark field images of two junctions, in a scanning transmission electron microscopy mode taken at the [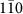] zone axis, are shown in Fig. 1c,d.

The triangular cross-section of GaN NWs provides locally planar interfaces on two facets^21^. The MgO layer is uniform and free of pinholes, thus ensuring high spin injection/detection efficiency^8^,^10^. The transmission electron microscopy analyses of the cross-sections (insets of Fig. 1c,d) identify the three facets as (001), (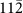) and (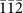). The selected area electron diffraction and high-resolution transmission electron microscopy analyses confirm that there are two uniquely different kinds of devices in our studies, characterized by the crystallographic planes that contact the ferromagnetic electrodes (Fig. 1c,d and Supplementary Fig. 3): the (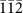) and (001) planes (Fig. 1c; type-C device) and the (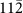) and (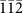) planes (Fig. 1d; type-D device).

Electrical measurements of a top gated GaN device with four electrodes showed the NW resistivity to be quite low, 1.7 and 1.6 mΩ cm at 1.8 and 300 K, respectively (Supplementary Note 1). Weak gate modulation, the temperature dependence of the resistance and low contact resistance were observed. These characteristics confirm that the GaN NWs are highly degenerate *n*-type semiconductors^22^ (Supplementary Fig. 4), behaviour that has been explained by the presence of nitrogen vacancies and/or oxygen impurities^23^.

### NLSV magnetoresistance measurements

**Figure 2:**
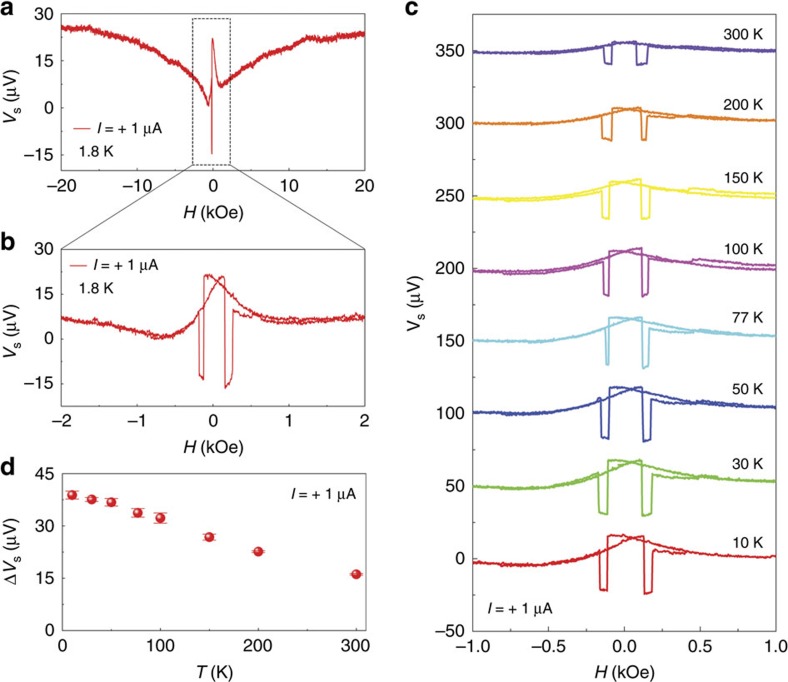
NLSV MR data. (**a**) Non-local MR measurement with field *H*_*y*_ applied along the *y* axis of device 01 (type-C device) at *T*=1.8 K. *I*=+1 μA. (**b**) Expanded field range near *H*_*y*_=0. (**c**) NLSV voltage dips Δ*V*_S_ were measured at several different temperatures with a current of +1 μA. (**d**) Temperature dependence of the amplitudes of Δ*V*_S_. The error bars in **d** indicate the s.d. from multiple measurements.

Data from a non-local magnetoresistance (MR) measurement with field *H*_*y*_ applied along the *y* axis of device 01 (type-C device) over a large field range are shown in Fig. 2a. Several independent effects of spin accumulation are identified and introduced. Although *H*_*y*_ is in the plane of the substrate, the injection and detection regions of the ferromagnetic films are in planes at angle ±60° to the *y* axis and magnetization anisotropies control the local magnetization orientations. The saturation magnetization of thin CoFeB films is 4π*M*_S_=11 kOe. A strong shape anisotropy forces the magnetization orientation M to lie in the film plane for field *H*_*y*_<<4π*M*_S_, whereas fields *H*_*y*_>4π*M*_S_ are sufficiently large that M always aligns parallel with *H*_*y*_. For |*H*_*y*_|>12 kOe (Fig. 2a), both M1 and M2 are aligned with *H* for all portions of the films. The injected spins also are aligned with *H*, there is no spin precession and the detector voltage measures the full, positive value of spin accumulation 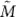.

For the range 2 kOe<|*H*_*y*_|<12 kOe, the in-plane shape anisotropy competes with the external field and the magnetization component in the plane of the film increases as |*H*_*y*_| decreases. The red trace in Fig. 2b shows the region −2 kOe<*H*_*y*_<2 kOe where the in-plane anisotropy dominates. Magnetizations M1 and M2 are in the film plane, but different portions of the injector and detector films in contact with the NW may have different vector orientations. In this field region, three separate features of spin accumulation are identified. First, narrow hysteretic dips are observed at |*H*_*y*_| ≈ 250 Oe, where M1 and M2 reverse orientations by 180°. Noting a baseline offset^24^ of about 5 Ω, the magnitude of the MR dip, Δ*R*_S_=2*R*_S_≈38 Ω at 1.8 K is consistent with the magnitude of 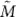 measured at |*H*_*y*_|=20 kOe: *R*_S_≈20 Ω. It follows that the local orientations of M1 and M2 are antiparallel during the re-orientation and, regardless of the unusual topography, measuring *R*_S_ using MR dips is a good representation of 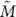. Second, data for up- and down-sweep traces in Fig. 2b are nearly identical for the field range |*H*_*y*_|> 250 Oe but show hysteresis for the range |*H*_*y*_|<250 Oe. Broad curves in this field range will be analysed using a model of spin accumulation and Hanle effect. Electron spin accumulation 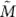 can transfer spin angular momentum to the lattice nuclei through an Overhauser coupling^25^,^26^,^27^,^28^,^29^,^30^,^31^. Nuclear spin polarization appears as an effective magnetic field, *H*_n_, that causes a hysteretic field shift of the Hanle line shape. Third, smooth and continuous traces are formed after adjusting the data to account for Overhauser shifts. These traces are matched with Hanle fitting functions below. The deduced spin relaxation times agree with the data and analysis in the narrow hysteretic dips. We use the Hanle data to show that type-C and type-D devices have very different spin transport characteristics: spin injection is robust at (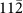) and (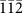) interfaces but is weak at the (001) interface.

### Temperature and channel length dependences of spin signal

We begin with a study of 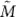 by analysing the MR dips. Non-local MR data are shown in Fig. 2c for a large temperature range, 10 K ≤ *T* ≤ 300 K. Our first significant result is a very large value of spin accumulation, Δ*R*_S_=38 Ω (1.8 K), which persists at room temperature, Δ*R*_S_=16 Ω (Fig. 2d). As the highly degenerate GaN NW has low room-temperature resistivity, the ratio of 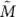 to channel resistance *R*_C_ is high, Δ*R*_S_/*R*_C_ ∼ 0.006 (0.003) at 1.8 K (300 K). In the temperature-dependent measurements of Δ*R*_S_, there is a possibility that the interfacial spin scattering may have played an important role in the analysis of NLSV signals^32^. To elucidate the effect in our system, we have investigated a temperature-dependent asymmetry between *R*_S_ at parallel and antiparallel configuration, indicating that the interfacial spin scattering effect is negligible even at room temperature in our device with a GaN NW (Supplementary Note 3). Spin accumulation is not usually observed in semiconductors at room temperature because of rapid spin relaxation^8^,^29^,^33^,^34^. The observation of large Δ*R*_S_ at room temperature in our GaN NWs implies long spin relaxation times, confirmed by the Hanle data presented below. The spin diffusion length *λ*_S_ was measured directly by fabricating a NLSV device (type-C device) with multiple ferromagnetic electrodes and several injector/detector separations, *L* (Supplementary Note 4). In the NLSV devices with multiple ferromagnetic electrodes, the spin absorption (spin sink) effect should be considered^35^,^36^. However, this effect cannot affect the estimate of spin transport characteristics in our devices, because the MgO tunnelling layer has high resistance (Supplementary Note 4). Measurements of Δ*R*_S_ (*L*) showed an exponential decrease exp(-*L*/*λ*_S_) with a fitted value of *λ*_S_=710±90 nm (300 K). The average polarization *P* of the ferromagnetic electrodes^37^ is found to be 8.9±0.5% (300 K). For the entire set of samples, *P* ranged from 9.4 to 13% (300 K; Supplementary Table 1).

### Hysteretic field shift of spin signal

The Overhauser coupling between spin accumulation and nuclear spins is described in Supplementary Note 5. A large Overhauser coupling of GaN NWs is related to the large spin accumulation in the confined geometry and highly efficient dynamic nuclear polarization of a Ga interstitial impurity^29^. By recognizing hysteretic shifts of field, by an amount ±*H*_n_, we combine sections of up- and down-sweeps to create smooth curves, Δ*R*_S_ (*H*).

### Hanle effect in NLSV MR

We analyse these magnetic field traces Δ*R*_S_ (*H*) using Hanle shape functions to fit the data. In most prior work with bulk and thin film samples, the non-magnetic channel and the ferromagnetic electrodes of the NLSV devices are coplanar^1^,^4^,^12^,^20^,^38^. NLSVs with GaN NW channels represent a specific device category with a geometry that is not coplanar. Each ferromagnetic electrode makes interfacial contact with two of the triangular faces and neither face is in the substrate plane. Each electrode may have one of a variety of local micromagnetic configurations and the injected spins generally will not have orientations that are coplanar with a detecting interface. Although we cannot know the details of the local magnetization orientations of the portions of spin injector and detector films in contact with the NWs, we show that a combination of absorptive and dispersive Hanle shape functions^39^ can fit any trace (Supplementary Note 6).

## Discussion

**Figure 3:**
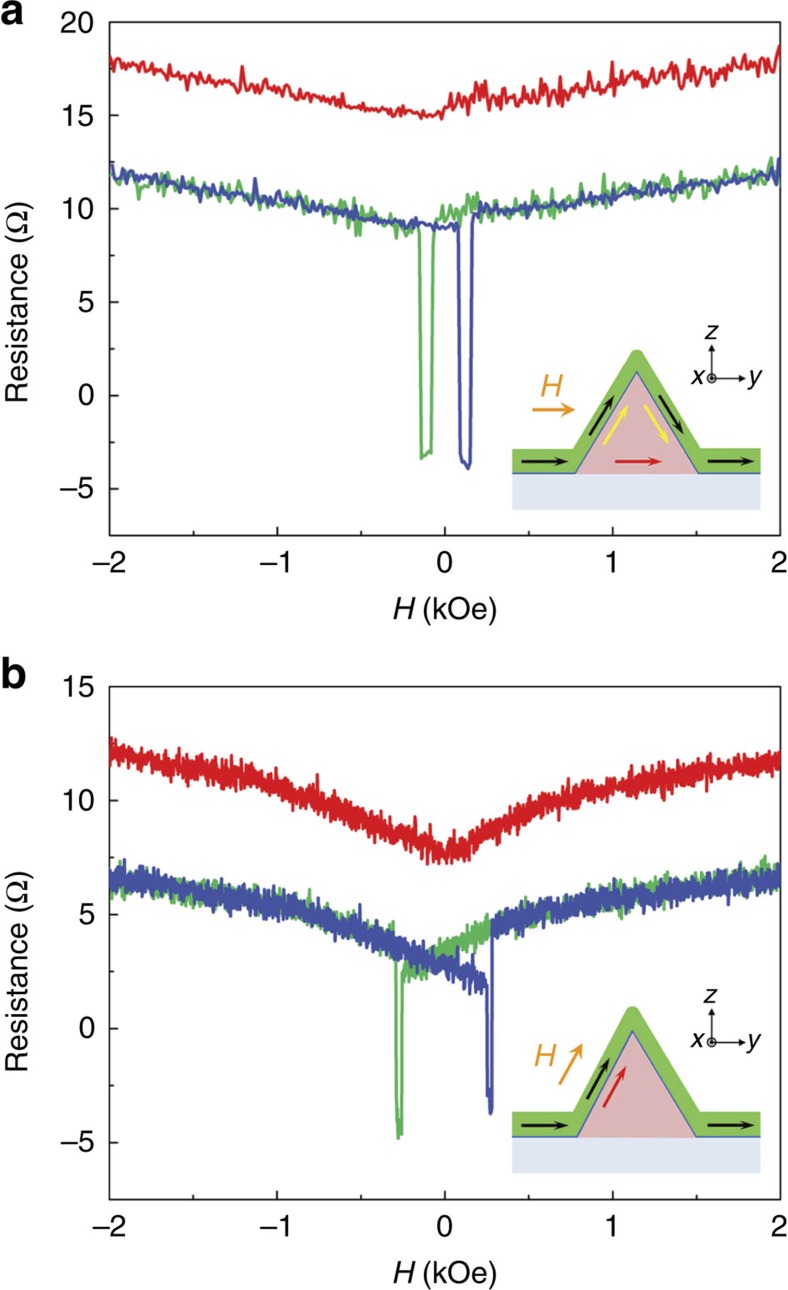
Spin injection of two typed devices at room temperature. (**a**) Nonlocal MR measurement *R*_S_ (*H*_*y*_) with field *H*_*y*_ applied along the *y* axis (inset) of device 02 (type-D). Green (blue) trace: field sweep down (up). Red trace: data are modified for Overhauser shifts and omission of dips, then shifted up for clarity. No Hanle feature is observed. (**b**) *R*_S_ (*H*) for device 03 (type-C), with *H* applied along an axis in the *y*–*z* plane at angle 30° to *z* (the inset). Green (blue) trace: field sweep down (up). Red trace data are modified for Overhauser shifts, omission of dips and shifted for clarity (Supplementary Note 4). *T*=300 K.

The inset of Fig. 3a shows an example in which the ferromagnetic electrode magnetization is continuous across the top of a type-D NW. Spin-polarized carriers injected at the left (right) face will be oriented in the *y*–*z* plane, along an axis 30° (−30°) to *z*. The resulting spin accumulation 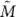 can be described as two populations in the *y*–*z* plane, each with a unique vector. In this case, there is equal spin injection from the two interfaces [(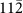) and (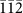)], the *z*-component of spin cancels and the net spin accumulation 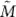 (red arrow in inset) is described as a vector along *y*.

Figure 3a shows Δ*R*_S_ (*H*_*y*_) for field applied along the *y*-axis. With 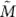 along *y*, there is no torque on the spins and no Hanle effect is observed. The usual MR dips are recorded and the Overhauser shift is negligible. When the dips are removed, a small, V-shaped background resistance is observed (red trace) and can be associated with magnetization M1 and M2 tipping slightly out of the film plane. These data confirm that left and right interfaces [(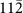) and (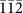)] inject spins with equal efficiency.

**Figure 4:**
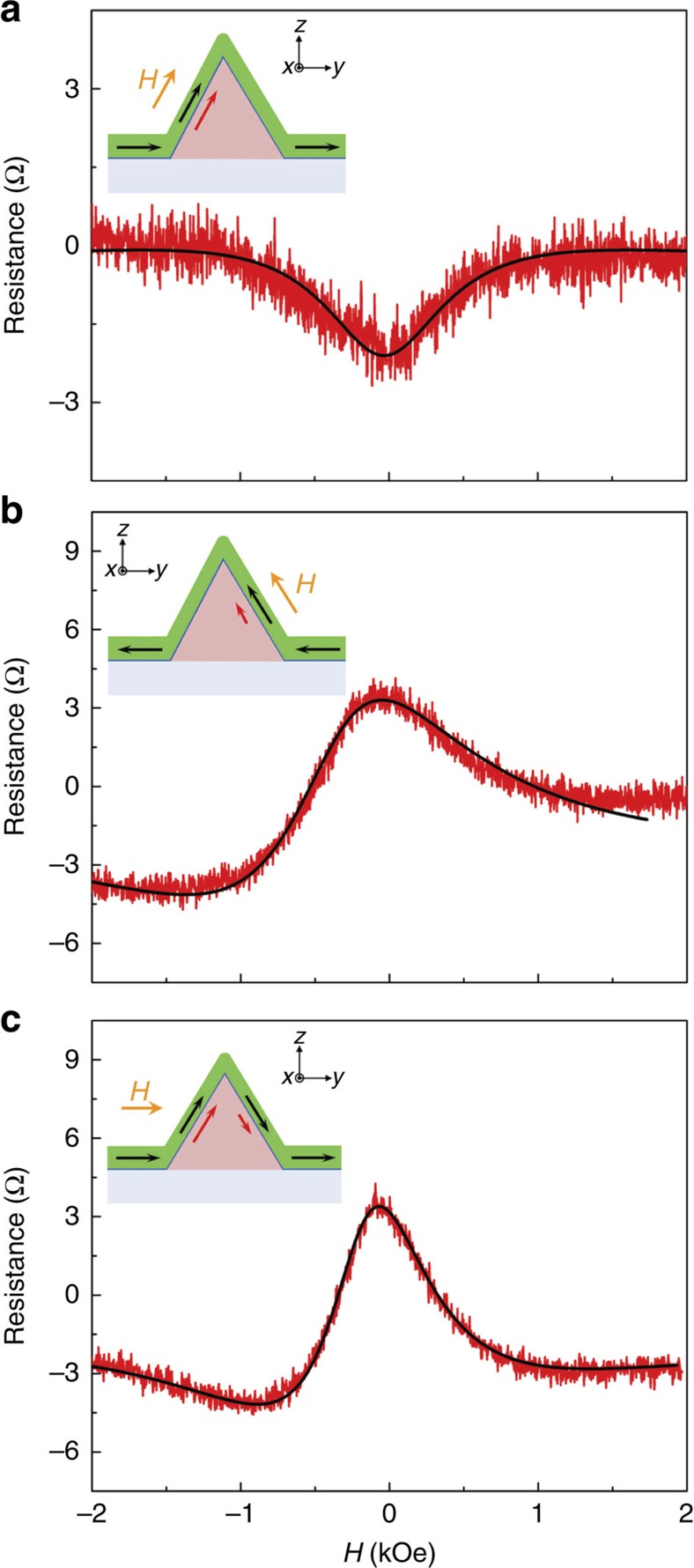
Difference in spin transport of crystallographic planes. All data sets are modified for Overhauser shifts of ±*H*_*n*_ and omission of MR dips (Supplementary Note 4). (**a**) *R*_S_ (*H*) for device 03 (type-C), with *H* applied along an axis in the *y*–*z* plane at angle 30° to *z* (refer to inset). The small baseliner resistance measured in Fig. 3a is subtracted from the red trace in Fig. 3b. The residual data are plotted and fit to a purely absorptive Hanle shape function. These data show that the Hanle effect derives from spins injected at the right (001) interface. (**b**) *H* is applied along an axis to 150° (refer to inset) and the trace is fit with a mixture of absorptive (40%) and dispersive (60%) Hanle shape functions. The Hanle effect derives from spins injected at the left (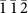) interface. (**c**) Field *H* is applied along the *y*-axis for device 01. The fit is for 60% absorptive and 40% dispersive components. Compare with data under the same conditions for a type-D device (red trace, Fig. 3a) where no Hanle effect is observed. All data were obtained at 300 K.

In a different case, field *H* is applied along an axis in the *y*-*z* plane and at an angle 30° to *z* (Fig. 3b, inset). No torque is applied to spins injected from the left interface, but spins injected from the right will show a Hanle effect. Figure 3b shows Δ*R*_S_ (*H*) for a type-C device along an axis in the *y*–*z* plane and *θ*=30° from *z*. The red trace has been modified to correct for Overhauser shifts and to omit the narrow dips. The resulting curve deviates from the red trace in Fig. 3a. To study the difference, we fit the V-shape trace (Fig. 3a) and subtract this background from the red trace in Fig. 3b. The residual plot is shown in Fig. 4a (note change of scale). The black line is a fit to the absorptive Hanle shape function^39^ (Supplementary Note 7). In our model of separate spin populations from the left and right interfaces, the magnetization orientations of the injecting and detecting ferromagnetic electrodes at the left interfaces, M1L and M2L, respectively, have dominant components along the 30° axis. These injected spins are oriented along the same axis as the magnetic field and never precess. For this type-C device, the observed Hanle curve must be the result of spin injection at the right (001) interface.

Figure 4b shows Δ*R*_S_ (*H*) with field *H* applied to the same device at an axis 150° to *z*. Here field *H* is parallel with the axis of spins injected from the right interface. These spins show no precession and no Hanle effect at the right (001) interface. The observed Hanle effect must derive from spin injection from the left (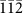) interface. The data are fit to a mix of dispersive (60%) and absorptive (40%) shape functions with amplitude of 5 Ω. Note that the Hanle feature that results from spin injection at the (001) interface is much smaller than the feature that results from spin injection at the (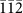) interface, an amplitude ratio of 0.4 for the data in Fig. 4a,b. The data in Fig. 4c show the Hanle feature when *H* is applied along the *y* axis (device 01, Fig. 2c). Field *H*_*y*_ is not collinear with the axes of spins injected from either interface and the Hanle curve differs from that in Fig. 4b. Comparison of Fig. 4c with the same experimental conditions for a type-D device (red trace, Fig. 3a) dramatically illustrates the very different spin transport characteristics of the (001), (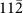) and (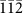) interfaces. These characteristics reproduced in our study of eleven type-C devices and six type-D devices. Examples of data and fits for other field configurations are shown in Supplementary Note 8. For a given device, fitting parameter *T*_2_ (spin relaxation time) showed little variation with temperature for *T*=1.8 and 300 K. For all fits, values of *T*_2_ were in the range 100–150 ps, which is comparable to the reported values^17^,^40^ (Supplementary Table 1).

These spin behaviours suggest that spin accumulation strongly depends on the interface where spin injection occurs. Wurtzite crystals exhibit non-zero spontaneous polarization resulted from the Ga- or N- face crystal plane of GaN. Transport properties of GaN are known to be largely dependent on the spontaneous polarization which is ten times larger than in conventional III–V semiconductor compounds^41^,^42^. The triangular geometry of our GaN NWs provides interfacial conditions that permit observation of the unique spin transport behaviour shown in Figs 3 and 4. Spontaneous polarization in GaN can cause electric fields up to 3 MV cm^−1^ (=0.3 V nm^−1^)^41^. One polar face at the (001) interface and two semi-polar faces at the (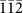) and (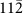) interfaces yield different magnitudes of electric field. Moreover, we speculate that the different interfaces, the (001) and (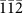) [or (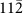)] planes, may alter the effect of the interface-specific spin filtering or the strength of the spin–orbit coupling^43^. A combination of these effects may cause the significant differences of spin injection at the (001) and (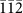) interfaces that result in the unusual spin accumulation observed in Figs 3 and 4.

In summary, we have studied electrically injected and detected spin transport in trigonal single-crystal GaN NWs and demonstrated robust spin injection and accumulation. The spin modulation resistance is large, Δ*R*_S_=16.1 Ω at room temperature. Because of the low NW resistivity (order of 10^−3^ Ω cm), the ratio of spin dependent modulation to channel resistance, *ΔR*_S_/*R*_C_∼10^−3^, is much higher than has been observed in other semiconductor materials. Remarkably, the room temperature spin transport parameters are comparable with, or larger than, values for aluminium (Al) films at cryogenic temperature: *λ*_S_ (GaN NW, 300 K)=710 nm∼*λ*_S_ (Al, 4 K)=800 nm and *T*_2_ (GaN NW, 300 K)=150 ps>*T*_2_ (Al, 4 K)=110 ps (ref. 44). These characteristics are promising for room temperature mesoscopic spintronic devices. A large Overhauser coupling between the conduction electron spin accumulation and the lattice nuclear spins was observed. Of greater interest, we demonstrated a large difference in the spin accumulation of different crystallographic planes. This opens possibilities to tune interfaces for optimized spin transport in mesoscopic devices.

## Methods

### NW growth

GaN NWs were synthesized in horizontal chemical vapour deposition system through vapour–liquid–solid mechanism using Au catalyst. Solid metallic Ga (purity 99.99%) was subsequently inserted in front of each substrate. The temperature of the furnace was increased at a rate of 50 °C min^−1^ to 850 °C. Flows of NH_3_ and H_2_ served as carrier and reactant gases at a rate 10 and 350 standard cubic centimetres per minute, respectively. The temperature was maintained for 120 min. and then cooled down to room temperature.

### NW-based device fabrication

A single GaN NW was picked up and transferred to a pre-patterned *p*^+^ Si substrate (*ρ*∼0.01 Ω cm) coated with 300 nm-thick of silicon oxide. The native oxide of the NW was removed with a hydrofluoric acid (deionized water/hydrofluoric acid (HF) 30:1 v/v) dip for 30 s. Electrical contacts (Ti/Au, 5/80 nm) were then formed by electron beam lithography and lift-off. A top gate structure consisting of an Al_2_O_3_ (30 nm) gate oxide and a Ti/Au (5/100 nm) top gate was formed on top of this NW using electron beam lithography followed by a lift-off process without wet etching (Supplementary Fig. 4). The fabrication of the NLSV devices was carried out with a two-step process. The first step was formation of the ferromagnetic spin injector and detector, and the second step was fabrication of the non-magnetic electrodes. In the first step, the surface oxide of the GaN NW was removed with a hydrofluoric acid dip and a 1.5 nm thick MgO tunnel barrier and 30 nm-thick CoFeB electrodes were subsequently deposited in a DC magnetron sputter system. The MgO layer is uniform and free of pinholes, ensuring high spin injection/detection efficiency^8^,^9^,^45^. Next, a Ta/Ru (5/5 nm) bilayer was deposited as a cap to prevent surface oxidation of the CoFeB. For the second step, a Ti/Au (5/100 nm) bilayer was deposited using DC magnetron sputtering after a brief Ar ion mill etch eliminated the oxide layer on the NW surface. All deposition procedures were performed *in*-*situ* without breaking vacuum. Electrical measurements of fabricated devices were carried out with a physical property measurement system (PPMS 9T, Quantum Design, Inc.) at stable temperatures in the range 1.8 K≤*T*≤300 K.

### Micromagnetic simulations

Micromagnetic simulations shown in Supplementary Note 6 were performed using the Object-Oriented MicroMagnetic Framwork developed by NIST^46^. The equilibrium magnetization configurations were obtained using a conjugate gradient algorithm with the Fletcher–Reeves method. The sample dimensions were 600 × 14,000 × 30 nm^3^ as the spin injector (F2) and 300 × 21,600 × 30 nm^3^ as the spin detector (F1) and the unit cell size was 3 × 3 × 3 nm^3^. The material parameters used are an exchange stiffness *A*_ex_=2.83 × 10^−11^ J m^−1^ and saturation magnetization *M*s=1.034 × 10^6^ A m^−1^.

### Data availability

The data that support the findings of this study are available from the corresponding authors on request.

## Additional information

**How to cite this article:** Park, T.-E. *et al*. Large spin accumulation and crystallographic dependence of spin transport in single crystal gallium nitride nanowires. *Nat. Commun.* **8,** 15722 doi: 10.1038/ncomms15722 (2017).

**Publisher's note:** Springer Nature remains neutral with regard to jurisdictional claims in published maps and institutional affiliations.

## Supplementary Material

Supplementary Information Supplementary Figures, Supplementary Table, Supplementary Notes and References
