# Supplementary material for: Large spin accumulation and crystallographic dependence of spin transport in single crystal gallium nitride nanowires
Source: Nat Commun. 2017 Jun 1;8:15722. doi: 10.1038/ncomms15722 (PMC5461503; doi:10.1038/ncomms15722)
Supplement: Supplementary Information — Supplementary Figures, Supplementary Table, Supplementary Notes and References [file ncomms15722-s1.pdf]

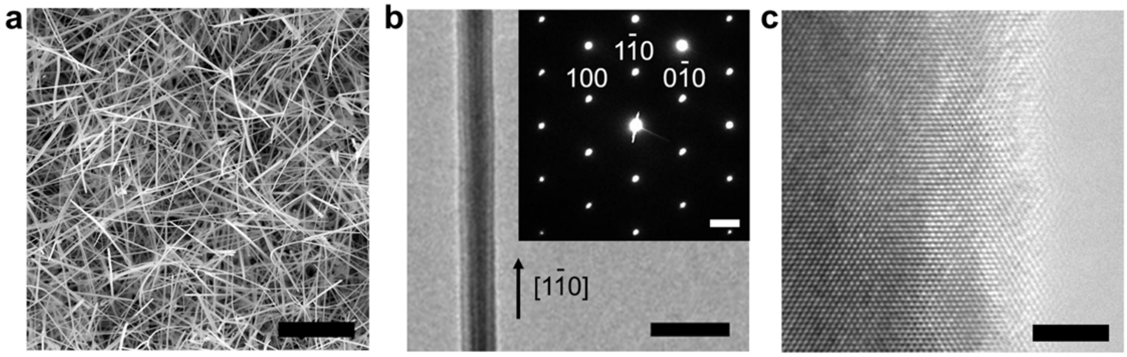

Supplementary Figure 1. Structural characterizations of gallium nitride nanowires. (a) A scanning electron microscopy (SEM) image of as-grown gallium nitride nanowires (GaN NWs) on a *c*-plane sapphire substrate (scale bar, 3 μm). **(b)** A transmission electron microscopy (TEM) image of the individual GaN NW (scale bar, 200 nm). The inset is the selected area electron diffraction (SAED) patterns recorded along [001] zone axis (scale bar, 2 nm^{-1}). **(c)** Lattice-resolved TEM image of the GaN NW (scale bar, 4 nm).

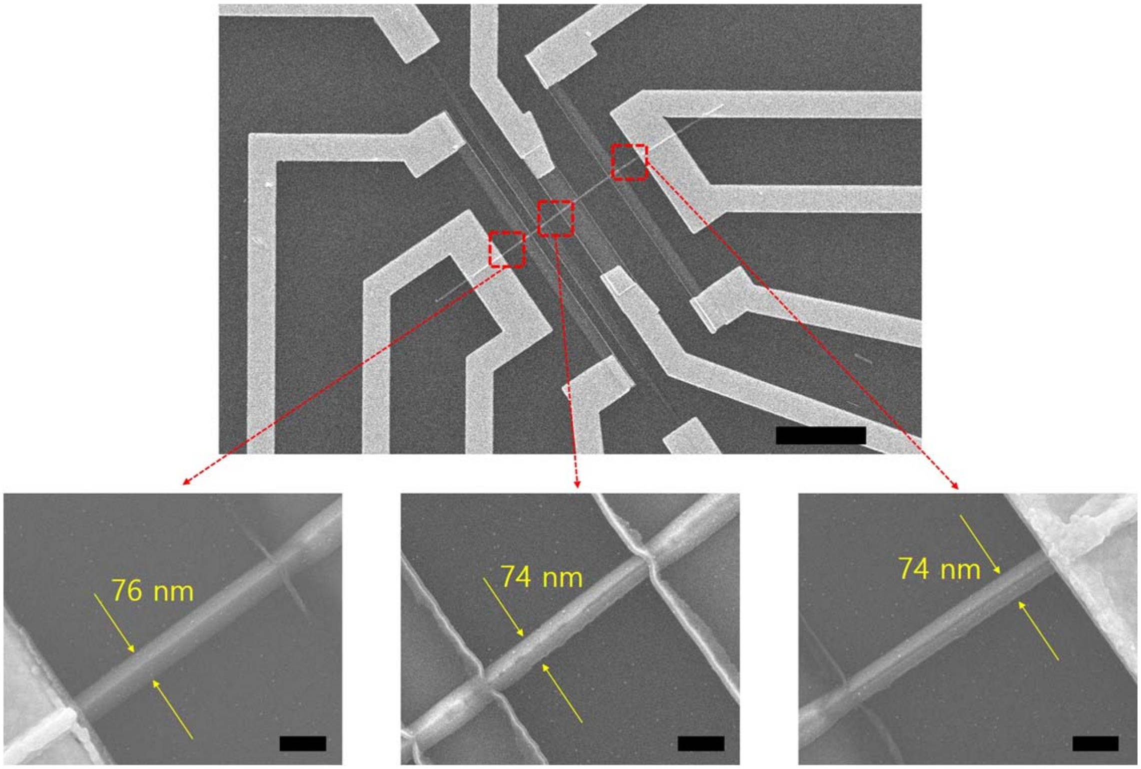

Supplementary Figure 2. Studied gallium nitride nanowire. A scanning electron microscopy (SEM) image of a typical nonlocal spin valve device composed of gallium nitride nanowire (GaN NW) with a scale bar of 3 μm. Detailed SEM images of the studied GaN NW (scale bars are 100 nm).

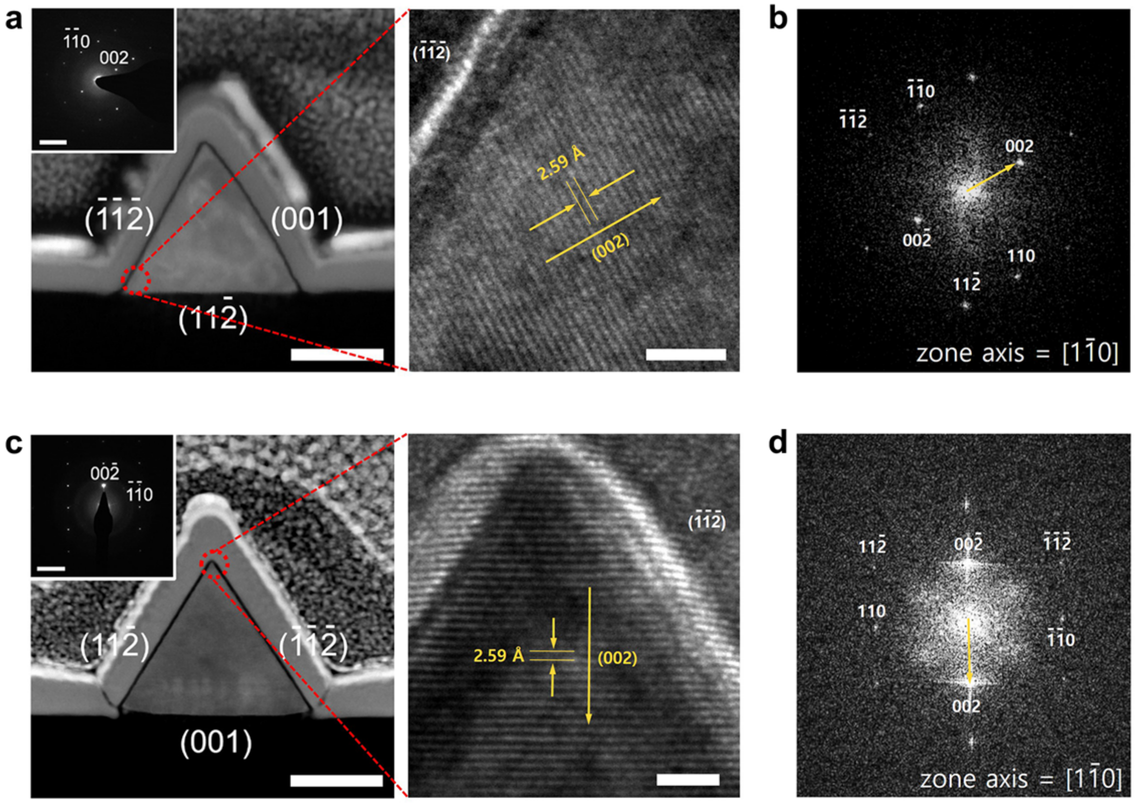

Supplementary Figure 3. High resolution transmission electron microscopy analyses. (a) and **(b)** high resolution transmission electron microscopy (HRTEM) image (scale bars in **(a)** are 40 and 2 nm, respectively) and corresponding fast Fourier transform (FFT) patterns of the type-C device in Fig. 1 **(c)**. **(c)** and **(d)** HRTEM image and corresponding FFT patterns of the type-D device in Fig. 1 **(d)** (scale bars in **(c)** are 40 and 2 nm, respectively). The scale bars in the insets of **(a)** and **(c)** are 5 nm^{-1}.

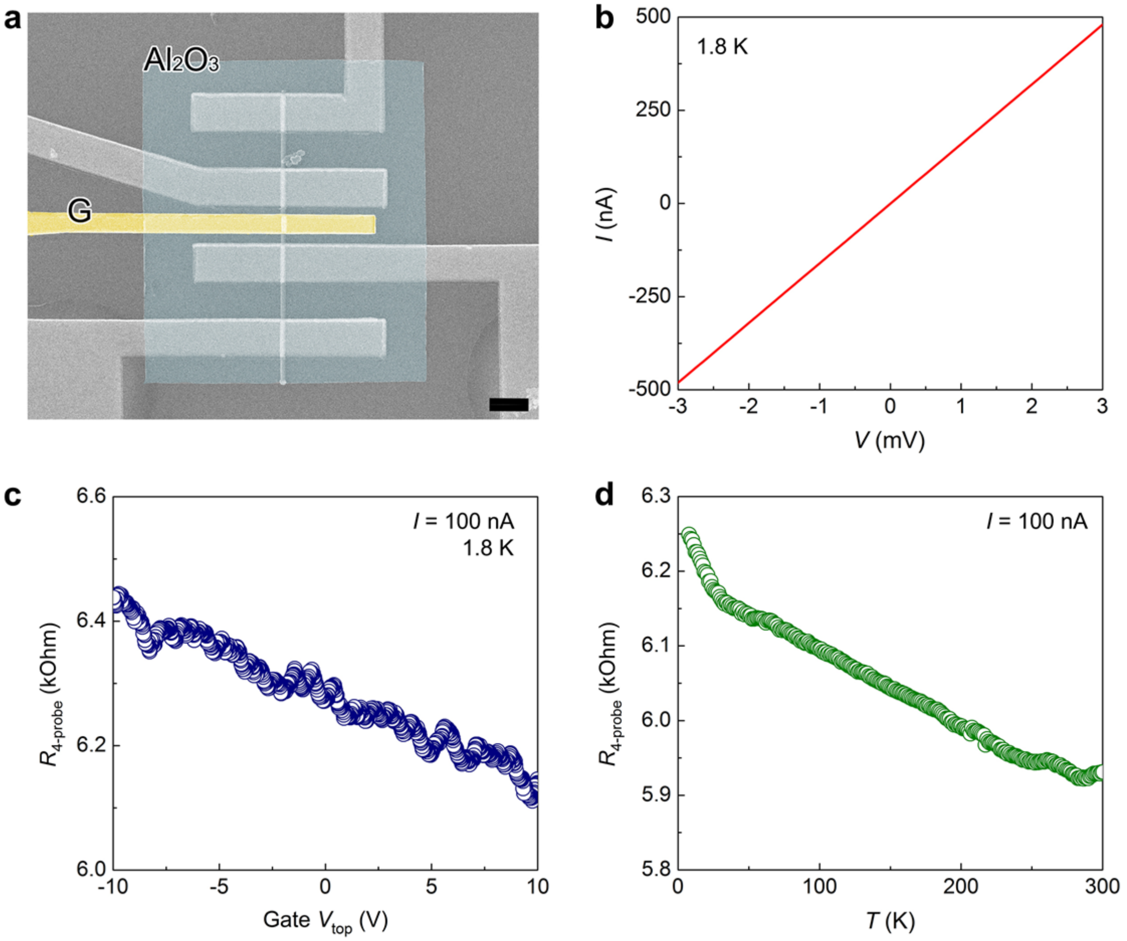

Supplementary Figure 4. Electrical properties of a gallium nitride nanowire. (a) A Scanning electron microscopy (SEM) image (with false colours) of a top gated gallium nitride nanowire (GaN NW) device and four electrodes with a scale bar of 1 μm. Blue: gate oxide (Al_2O_3). Yellow: gate. **(b)** *I*-*V* characteristics of the GaN NW measured by four-probe geometry. $T = 1.8$ K; $V_G = 0$ V. **(c)** Resistance of the NW as a function of applied gate voltage, $V_G = V_{top}$, showing *n*-type transport behavior. $T = 1.8$ K. **(d)** Resistance of the NW as a function of temperature measured with a bias current of 100 nA. $V_G = 0$ V.

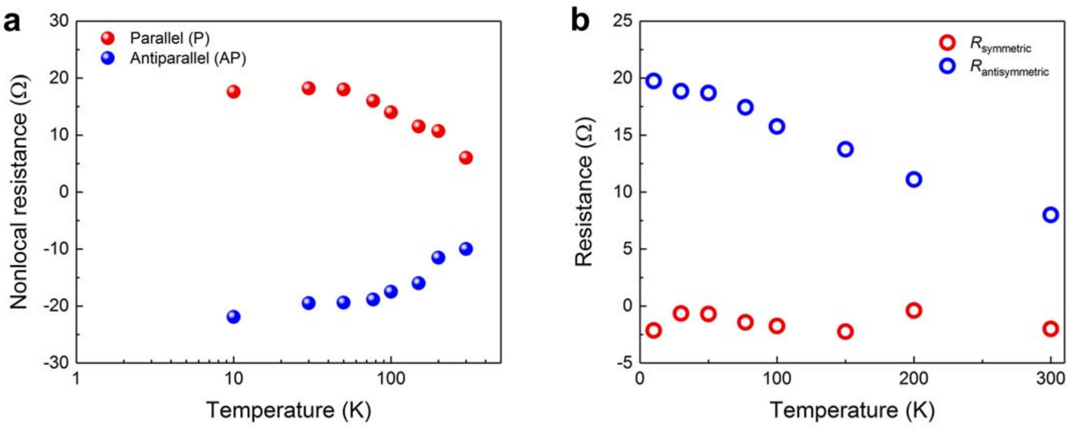

Supplementary Figure 5. Temperature dependence of the nonlocal spin valve signal. (a) Temperature dependence of the nonlocal spin valve resistance (R_S) for the parallel (red circles) and antiparallel (blue circles) configurations (device 01; type-C device). **(b)** Temperature dependence of the symmetric and antisymmetric components of R_S.

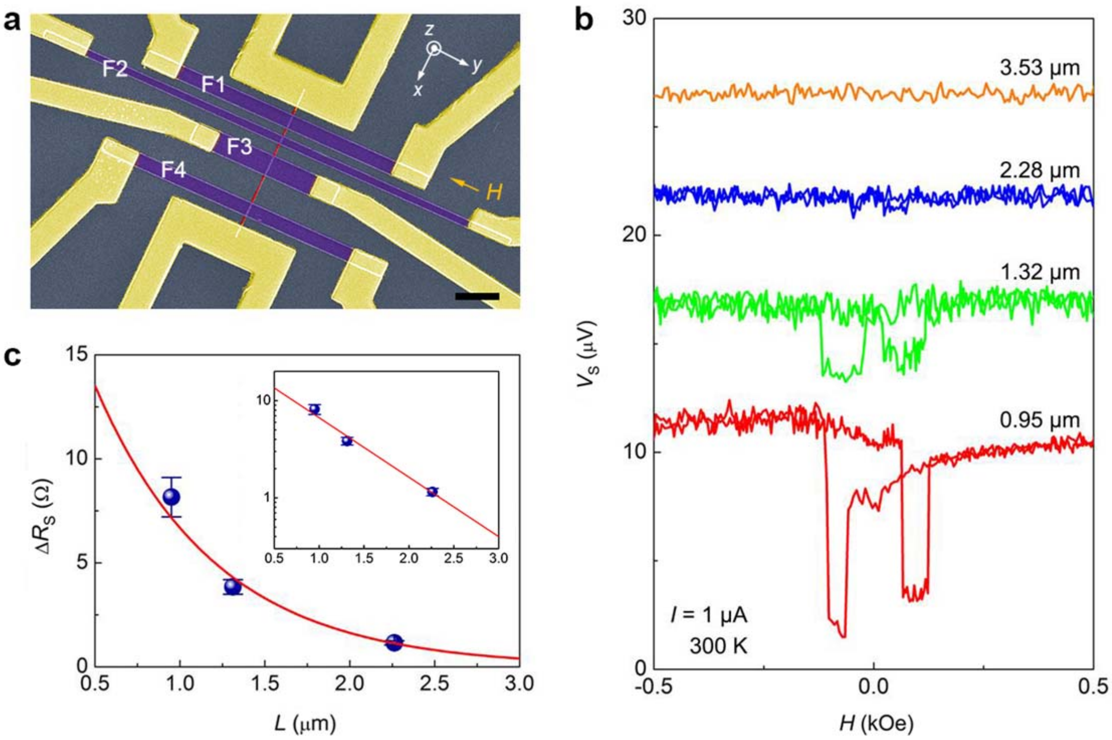

Supplementary Figure 6. Channel length dependence of nonlocal spin signal. (a) False-coloured scanning electron microscopy (SEM) image of a device with an array of four CoFeB electrodes (F1~F4) contacting a gallium nitride nanowire (GaN NW) used to measure the spin diffusion length λ_S and spin injection/detection efficiency *P*. The scale bar is 2 μm. **(b)** The nonlocal spin valve (NLSV) voltage were measured at different separation *L* (centre-to-centre distance) ferromagnetic injector and detector at room temperature: 0.95 μm (between F1 and F2), 1.32 μm (between F2 and F3), 2.28 μm (between F1 and F3), and 3.53 μm (between F2 and F4). The data for L = 0.95 μm are plotted without any offset and show true baseline voltage. The other plots are shifted for clarity. (c) The NLSV resistance change ($\Delta R_S = \Delta V_S / I$) on the centre-to-centre separation *L*. The inset shows a semilog plot of ΔR_S as a function of *L*. The red solid line is a fit to Supplementary Eq. 7. We measured the NLSV voltages with I = +1 μA with the magnetic field applied along the *y*-axis. T = 300 K. The error bars in **(c)** indicate the standard deviation from multiple measurements.

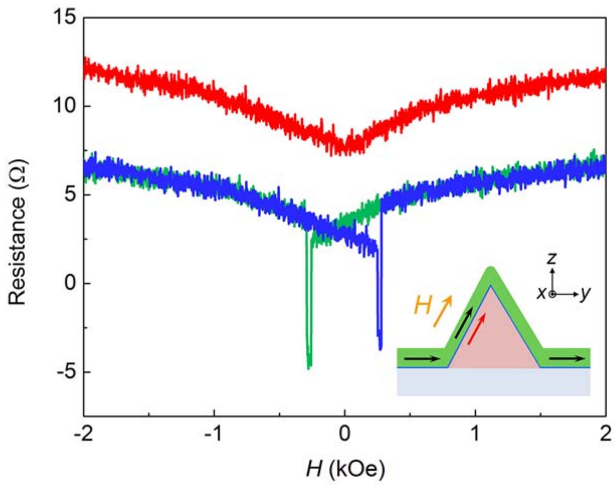

Supplementary Figure 7. Field shifts caused by Overhauser coupling. Nonlocal spin valve (NLSV) resistance is measured with field H applied in the y-z plane an axis 30° from z. Device 03 (type-C device). T = 300 K. Blue (green): field sweep up (down), raw data. Raw data for $H \leq 250$ Oe (blue trace) are shifted by $H_n = -250$ Oe and combined with raw data for $H \geq -250$ Oe (green trace; shifted by $H_n = +250$ Oe) to form modified trace (red) that is analyzed for the Hanle effect. The red trace is offset for clarity. Inset: Cross-section sketch of gallium nitride nanowire and CoFeB electrode, showing orientations of applied field (dark yellow arrow), magnetization (black arrows) and component of $\widetilde{M}$ that's injected from the left interface (red arrow).

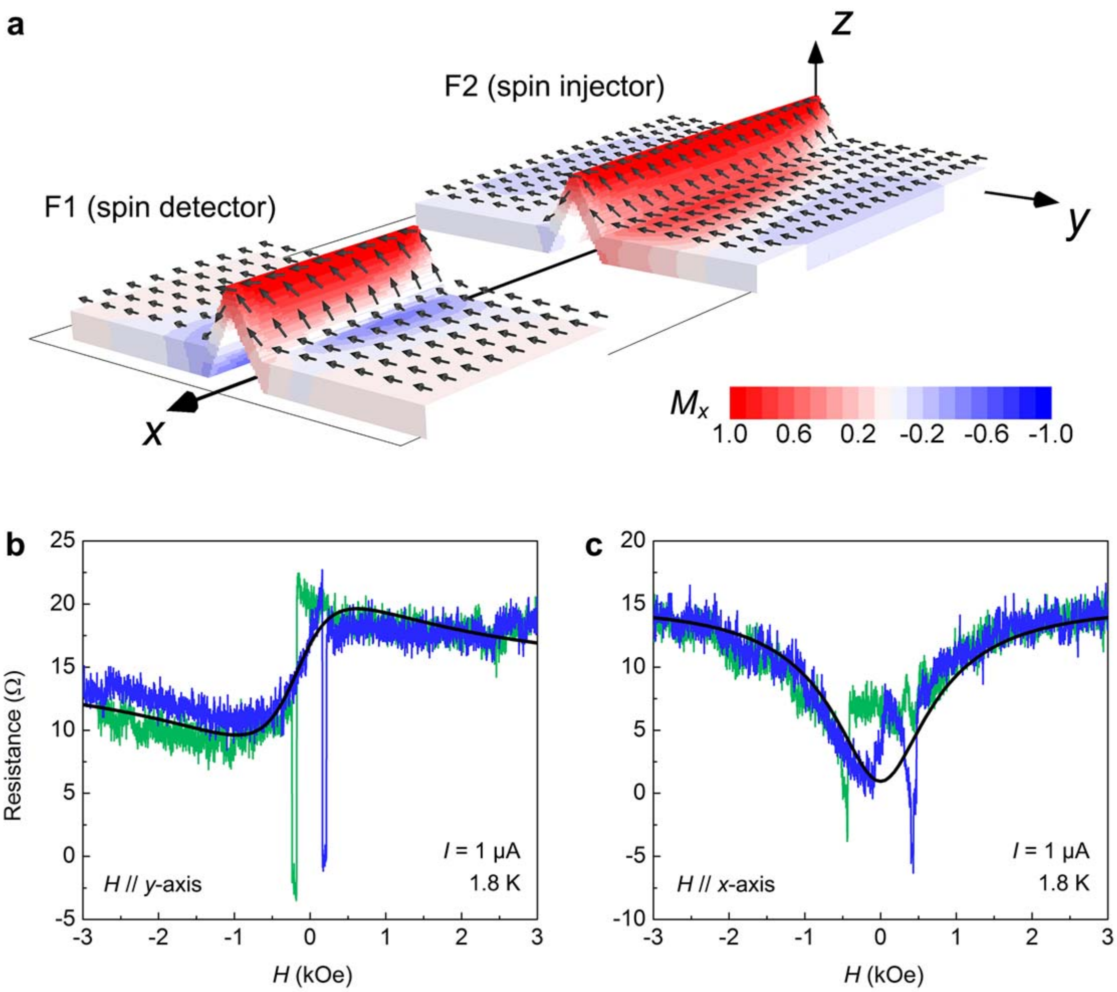

Supplementary Figure 8. Local injector/detector magnetization orientations. (a) Schematic diagram of the spin injector and detector showing a micromagnetic simulation of the equilibrium magnetic configuration in the remanant state. The small arrows represent the orientations of the local magnetizations in the CoFeB electrodes. Magnetization components along the *x*-axis, which are mainly converged at the top of the spin injector and detector, are normalized and represented by red (+*x*) and blue (−*x*) shading. **(b)** and **(c)** The measured nonlocal magnetoresistance with $I = +1$ μA at T = 1.8 K when magnetic field H is applied along the *y*-axis **(b)** and the *x*-axis **(c)**. Black lines represent the Hanle fits to the data with the same parameter, B0 = 600 Oe, and dispersive **(b)** or absorptive **(c)** shapes. $I = +1$ μA. Green (blue) symbols: field sweep down (up).

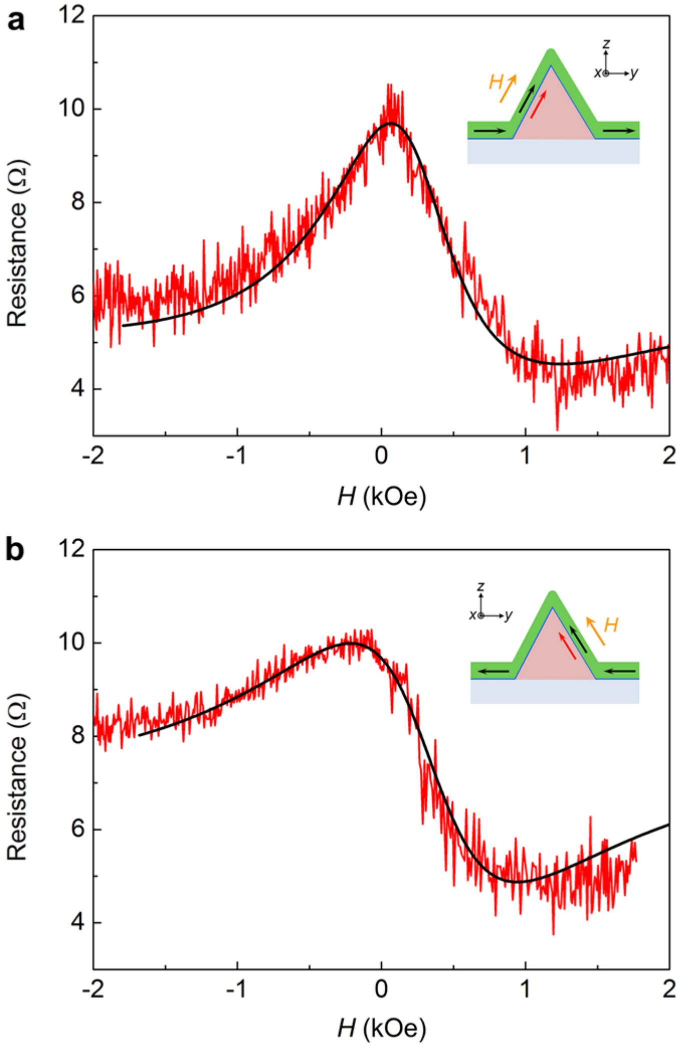

Supplementary Figure 9. **Angular dependence of the spin signals from type-D device. (a)** Angular dependence of the nonlocal spin valve magnetoresistance measured from a type-D device with $I = +1$ μA at room temperature. Raw data have been modified for Overhauser shifts and fit to Hanle shape functions. **(a)** H at 30° and **(b)** H at 150°. Insets show cross-section of sample and field orientations. Parameters of fits are given in Supplementary Table 1.

Device (id)	type	field (angle)	Temp (K)	Hanle fit	B0 (Oe)	T_2 (psec)	L0 (μm)	λ_S (nm)	ΔR_s (Ω)	P (%)
Fig.4a	C	30°	300	absorb dip	450	130	1.0	1000	8.8	9.4
Fig.4b	C	150°	300	0.4+0.6	600	98	1.0	1000	10.5	10
Fig.4c	C	90° (y)	300	0.6+0.4	390	150	1.3	770	16	13
n.a. (not shown)	C	90° (y)	1.8	0.55+0.45	360	160	1.3	770	34	18.5
Fig.3a	D	90° (y)	300	no Hanle	n.a.				12	11
Fig.9a (Supplementary)	D	30°	300	0.59-0.41	470	125	1.0	1000	12	11
Fig.9b (Supplementary)	D	150°	300	0.09-0.91	580	105	1.0	1000	12	11

Supplementary Table 1 | Examples of parameters derived from fits. Hanle fit refers to a linear combination of the two shape functions and gives fractional portions of the absorptive and dispersive components in the format C1 + C2. B0 and L0 are parameter values chosen to give the best fits (refer to Supplementary Note 7 and Supplementary Eqs. 4 and 5). Spin diffusion length λ_S is $\lambda_S = L$ / L0 with L = 1.0 μm. The magnetoresistance ΔR_S is measured from the narrow dips associated with M1 and M2 antiparallel.

Supplementary Note 1. Structural and transport characterizations of gallium nitride nanowires.

Here we provide the structural characterizations of the gallium nitride nanowire (GaN NW) samples. In Supplementary Fig. 1, we first exhibit the structural analysis of our NWs. To provide the information on the thickness/diameter uniformity along the entire wire, which is crucial for uniform spin transport, we also show the detailed scanning electron microscopy (SEM) images in Supplementary Fig. 2. Supplementary Fig. 2 reveals that our NW has a high uniformity with an average width of ~ 75 nm and a diameter variation within only a few nm. Moreover, to further identify the lattice planes of our GaN NWs, we have performed a high resolution transmission electron microscope (HRTEM) in Supplementary Fig. 3. In agreement with the selected area electron diffraction (SAED) study in Figs. 1c and 1d, the cross-section HRTEM analysis showed the presence of the $(\bar{1}\bar{1}\bar{2})$ and (001) planes in the type-C device and the $(11\bar{2})$ and $(\bar{1}\bar{1}\bar{2})$ planes in the type-D device, as shown in Supplementary Fig. 3. A SEM image of a top gated device is shown in Supplementary Fig. 4a. This device was used for the electrical transport measurements of a GaN NW shown in Supplementary Figs. 4b, 4c, and 4d.

Supplementary Note 2. Interface control for good spin injection to gallium nitride nanowire.

The criteria for mediating the conductance mismatch at a ferromagnetic metal/ semiconductor interface of area A is that the interface resistance r_I must be comparable with, or larger than, the unit resistance r_F or r_S of ferromagnetic or semiconducting materials, each with area A and thickness equal to a spin diffusion length, λ_F or λ_S[1–3]. The interface resistance r_I was measured to lie in the range 14.3 $\pm$ 1.4 kΩ. Using the measured spin diffusion length (see Supplementary Note 3), λ_S = 0.71 μm, the value r_S (1.8 K) is calculated to be r_S = 5.1 kΩ. The spin diffusion length in CoFeB is roughly 5 nm, and we estimated $r_F \sim 0.005$ Ω. Thus, the condition for robust spin injection, r_S and $r_F \leq r_I$ is satisfied.

Supplementary Note 3. Analysis on the temperature-dependent interfacial spin scattering.

The nonlocal spin valve (NLSV) signal (V_S or $R_S = V_S / I$) between parallel and antiparallel configurations could have a strong temperature dependence as studied in Ref. [4]. In these earlier studies, it has been proved that the temperature-dependent asymmetry in NLSV signal is directly related to the temperature-dependent interfacial scattering. Gazon *et al.* have shown that both the symmetric and the antisymmetric combination of R_S can provide information about interfacial spin scattering[4],

$$R_{\text{symmetric, antisymmetric}} = [R_S\,(\text{parallel}) \pm R_S\,(\text{antiparallel})] / 2.$$

To elucidate this effect in our system, we have investigated the temperature dependence of NLSV signals for the parallel (red circles in Supplementary Fig. 5a) and antiparallel (blue circles in Supplementary Fig. 5b) configurations. The temperature

dependences of $R_{\text{symmetric}}$ and $R_{\text{antisymmetric}}$ are plotted in Supplementary Fig. 5b. Note that $R_{\text{symmetric}}$ remains almost constant in this temperature range (10 K $\leq T \leq$ 300 K) while $R_{\text{antisymmetric}}$ decreases linearly with increasing T. Importantly, this weak temperature dependence of $R_{\text{symmetric}}$ reveals that the interfacial spin scattering effect is negligible even at room temperature in our NLSV devices with a GaN NW.

<u>Supplementary Note 4. Nonlocal spin valve measurements of a single nanowire with multi ferromagnetic electrodes.</u>

The NLSV signal is sensitive to the resistivity ρ of the channel, spin injection/detection polarization P, and the interface properties. We determine the spin diffusion length from measurements of the spin accumulation at variable distance, ΔV_{S} (L) or ΔR_{S} (L), using a GaN NW sample with multiple ferromagnetic probes (Supplementary Fig. 6a). Four separations ($L_{1\text{-}4}$) are used for measurements; injecting spin polarized electrons at F1 or F2 while using F2, F3 or F4 for detection. The centre-to-centre distances are 0.95 μm (between F1 and F2), 1.32 μm (between F2 and F3), 2.28 μm (between F1 and F3), and 3.53 μm (between F2 and F4). Especially for the cases when L = 2.28 μm and L = 3.53 μm, where there exist additional ferromagnetic electrodes between the injector and detector, the spin absorption effect (also called the spin sink effect) at the additional electrode should be considered. In earlier investigations, it has been shown that the spin absorption effect is strongly dependent on the ratio of the spin resistance of channel (R_{Ch}^{S}) to the spin resistance of the addition electrode (R_{El}^{S})[5,6]. It has also been proved that the spin absorption by

additional electrodes is only allowed when $R_{\mathrm{El}}^{\mathrm{S}} \ll R_{\mathrm{Ch}}^{\mathrm{S}}$ whereas the absorption is prevented when $R_{\mathrm{El}}^{\mathrm{S}} \gg R_{\mathrm{Ch}}^{\mathrm{S}}$. For instance, if we used *metallic* contacts between a channel and additional ferromagnetic electrodes, the spin sink effect would be significant due to the relatively small spin resistance at the contacts, $R_{\mathrm{El}}^{\mathrm{S}} \ll R_{\mathrm{Ch}}^{\mathrm{S}}$. However, in our study, the contacts between a GaN NW and ferromagnetic electrodes are non-metallic because they include MgO tunneling barriers. Therefore, this high interface tunnel resistance, resulting in $R_{\mathrm{El}}^{\mathrm{S}} \gg R_{\mathrm{Ch}}^{\mathrm{S}}$, prevents spin absorption into the additional ferromagnetic electrodes in our measurements.

The observed magnitude of the voltage change (ΔV_{S}) in the NLSV of Supplementary Fig. 6a exponentially decreased as L increased from 0.95 to 2.28 μm and no ΔV_{S} was observed at L = 3.53 μm (Supplementary Fig. 6b). The spin diffusion length and spin injection/detection polarization can be readily estimated by the channel length dependence, ΔR_{S} (L). The fit to Supplementary Eq. 7 is shown as the red curve in Supplementary Fig. 6c and gives the value λ_{S} = 710 $\pm$ 90 nm at room temperature. From Supplementary Eq. 7 (See Supplementary Note 7), the spin injection/detection polarization is P = 0.089 $\pm$ 0.005 at room temperature.

Supplementary Note 5. Overhauser coupling and effective nuclear field H_{n}.

We describe how hysteretic shifts in the data, near H = 0, are explained by an Overhauser coupling and an associated effective nuclear field (the Overhause field of magnitude H_{n}) that acts on the conduction electrons. To detect the nuclear field, oblique

Hanle effect measurements have been widely used[7–13]. In the NLSV magnetoresistance (MR) measurements of our GaN devices, the oblique external magnetic field is applied to the orientation of electron spins in a GaN NW because the injector and detector ferromagentic films are in planes at $\pm 60°$ to the plane of the substrate. Therefore, it is possible to observe the Overhasuer field through our NLSV measurements.

The green and blue traces in Supplementary Fig. 7 show raw data for down- and up-field sweeps, respectively, of device 03 (type-C, 300 K). Field H is applied along an axis in the y-z plane and $\theta = 30°$ from z (refer to inset). The green and blue traces overlap identically for the field ranges $| H_y | > 250$ Oe, but show hysteresis for the range $| H_y | < 250$ Oe. We describe the case of spins injected at the $(\bar{1}\bar{1}\bar{2})$ interface (left interface) which dominates spin transport. Consider first the green trace. For the field range from +2000 Oe to -250 Oe, the injector magnetization at the left interface is at a 30° angle (refer to inset) and the injected spins have orientation shown with the red arrow. The conduction electron spin accumulation couples with the nuclei in the lattice and the nuclear spins become polarized. The nuclear polarization appears as an effective magnetic field $+H_n$ and the net effective field that acts on the conduction electrons is $H_{net} = H_{external} + H_n$. In the range 250 Oe $\leq H \leq$ 2100 Oe, the injector magnetization at the left interface has orientation +30° for both up- and down-sweeps and the green and blue traces are the same. For the green trace, the effective nuclear field has this value also for the field range from +250 Oe to -250 Oe. At $H = -(250 - \delta H)$, the spin resistance changes to the value at field $H_{net} = -250$ Oe $+ H_n$. In the range $H = -250 \pm \delta H$ Oe, both injector and detector magnetizations reorient by 180° to produce the narrow MR dips. The orientation at the left interface now is at a 210° angle. The effective nuclear field changes to $-H_n$ and the spin resistance changes to the value at $H_{net} = -250$ Oe $- H_n$.

A corresponding process occurs with the blue trace. For H increasing from -2000 Oe to about +250 Oe the magnetization at the injector interface is oriented with direction 210°. The nuclear polarization appears as an effective magnetic field, $-H_n$. At $H = 250 - \delta H$ Oe, the net magnetic field in the GaN NW is H_{net} = 250 Oe $- H_n$. At $H = 250 \pm \delta H$ Oe, the magnetizations of the injector and detector films reverse, producing the narrow MR dip. Spins with the opposite orientation are injected, and the effective magnetic field has the opposite sign, $+H_n$. To correct for the nuclear fields, the green and blue traces are split at the dips, and the separate portions are recombined to create a smooth curve (Supplementary Fig. 7, red trace). The curves now join smoothly at $H = 0$ and we deduce that $| H_n |$ = 250 Oe. This technique is used in all our data sets and is justified, in each case, because the resulting trace is a smooth curve that is matched to a Hanle fitting function. For the modified trace in Supplementary Fig. 7, the fitting procedure is described in the text and the results are shown in Fig. 3b.

It was reported that the effective nuclear field can decrease rapidly with increasing temperature, following a T^2 dependence[12]. Figure 2, however, shows the hysteretic shifts in GaN NWs decrease with weaker temperature dependence. The deviation found in Fig. 2c may arise from the high impurity densities of our GaN NWs (heavily doped *n*-type NWs) and strong spin polarization of conduction-band electrons in the confined geometry via electrical spin injection. Large spin accumulation and hyperfine coupling can facilitate efficient dynamic nuclear polarization (DNP) of Ga interstitial impurities in a GaN NW at room temperature[14].

Supplementary Note 6. Plane dependent spin transport in a trigonal gallium nitride nanowires and micromagnetic simulations.

A unique feature of the NLSV MR data in our study is the presence of a Hanle effect observed in most orientations of external field, including the case where *H* is in the *x*-*y* plane and along the long axis of F1 and F2 (*y*-axis). Our GaN NWs with the triangular cross-sections present a unique case. When the spin polarized electrons tunnel from the CoFeB into the NW, the injected spins are parallel with the local magnetization orientation of the CoFeB, but the ferromagnetic electrodes on top of the NW have a non-planar topography (Supplementary Fig. 8a). This distortion affects the local magnetization of the portion of the CoFeB film in contact with the NW. The orientation of the injected spins and relative magnetizations of both CoFeB electrodes (spin injector and detector) are affected, simultaneously. In a conventional NLSV device having planar ferromagnetic electrodes, there is a uniaxial shape anisotropy along the film axis (y-axis). A magnetic field H_y controls switching between uniformly parallel or antiparallel states. In our GaN NW devices, portions of the CoFeB electrodes have local magnetizations that differ from the direction of the applied field.

This model is tested by simulating the local magnetization of distorted portions of the CoFeB electrodes using the Object-Oriented MicroMagnetic Framework (OOMMF)[15]. Supplementary Fig. 8a shows simulated equilibrium magnetization configurations of both spin injector and detector in the remanant state for a history of magnetization saturation along the *y*-axis and then reduction of field H_y to zero. The small arrows represent the orientations of the local magnetizations in the CoFeB electrodes located near the trigonal GaN NW. The

magnetizations of both the spin injector and detector are mostly in-plane and aligned along the y-axis. However, magnetization components along the transverse direction, represented by red ($+x$) and blue ($-x$) shading, are developed in the portions of the CoFeB films on or near the NW (Supplementary Fig. 8a).

The evolution of the x-component of magnetization can be understood by considering the magneto-static field inside the CoFeB electrode. This strong component M_x spreads out to the lateral facets of the NW and plays a major role in spin dependent transport detected by the NLSV measurement. It is noted that the x-component magnetization of the spin injector is much enhanced due to the large effective shape anisotropy along x-axis, which is induced by the larger width of spin injector (600 nm) compared with the smaller width of the spin detector (300 nm). Moreover, the local magnetizations of the spin injector and detector on the NW will not be aligned with each other. Instead, the local magnetization orientations are described by relative angle α. If the magnetizations of the spin injector and detector are aligned either parallel or antiparallel, one expects the absorptive Hanle shape when the magnetic field is applied perpendicular to the orientation of the injected spins. On the other hand, when the magnetizations of the electrodes are mutually perpendicular, the dispersive Hanle curve appears (see Supplementary Note 7)[16]. For arbitrary angle α the Hanle data will be a mixture of dispersive and absorptive[16]. Based on our understanding of the local magnetizations of both spin injector and detector, we analysed nonlocal MR data for external magnetic field applied along the y- and x-axis (Supplementary Figs. 8b and 8c). We observed dominantly dispersive (Supplementary Fig. 8b) and absorptive Hanle signals (Supplementary Fig. 8c) in NLSV measurements when the magnetic field was applied along y- and x-axis, respectively. Fits for both field orientations used the same parameter, B0 = 600 Oe. Note that

bias current $I = +1$ μA is used and the narrow dips that are expected in regions with M1 and M2 antiparallel appear in both Supplementary Figs. 8b and 8c. These Hanle fits imply that the relative orientations of the magnetization angle α should be nearly 90° and 0° (or 180°) when the magnetic field is applied along *y*- and *x*-axis, respectively.

S7. Analysis of Hanle data

In the NLSV MR measurements, the correct fitting functions for Hanle data are solutions to the Bloch equations with a one-dimensional diffusion term[16]. These solutions are expressed using two reduced variables. The first is:

$$\mathrm{b} \equiv \gamma B \mathrm{T}_2 = \frac{H}{\mathrm{B0}} \qquad (1)$$

$$\mathrm{B0} = H_{\mathrm{hwhm}} = \frac{1}{\gamma \mathrm{T}_2} \qquad (2)$$

where γ is the gyromagnetic ratio, H is the external magnetic field, H_{hwhm} is the half-width at half maximum, and T_2 is the spin relaxation time.

The second is

$$l \equiv \frac{L}{\sqrt{D\mathrm{T}_2}} = \frac{L}{\lambda_s} = \mathrm{L0} \qquad (3)$$

where L is the centre-to-centre distance between the spin injector and detector and λ_{S} is the spin diffusion length.

The Hanle fitting functions are expressed as:

$$G1 = \frac{1}{f\,(\mathrm{b})}\left[\sqrt{1+f(\mathrm{b})}\cos\left(\frac{l\mathrm{b}}{\sqrt{1+f(\mathrm{b})}}\right) - \frac{\mathrm{b}}{\sqrt{1+f(\mathrm{b})}}\sin\left(\frac{l\mathrm{b}}{\sqrt{1+f(\mathrm{b})}}\right)\right] e^{-l\sqrt{1+f(\mathrm{b})}} \qquad (4)$$

$$G2 = \frac{1}{f\,(\mathrm{b})}\left[\frac{\mathrm{b}}{\sqrt{1+f(\mathrm{b})}}\cos\left(\frac{l\mathrm{b}}{\sqrt{1+f(\mathrm{b})}}\right) + \sqrt{1+f(\mathrm{b})}\sin\left(\frac{l\mathrm{b}}{\sqrt{1+f(\mathrm{b})}}\right)\right] e^{-l\sqrt{1+f(\mathrm{b})}} \qquad (5)$$

where $f\,(\mathrm{b}) = (1 + \mathrm{b}^2)^{1/2}$. Here, G1 represents the absorptive shape function that arises when magnetizations M1 and M2 of injector and detector are either parallel (G1 positive) or antiparallel (G1 negative) and G2 is the shape function for the case when *M*1 and *M*2 have perpendicular orientation[16].

A general fitting function G3 is a linear combination of G1 and G2 with fractional contributions C1 and C2, respectively:

$$G3 = C1 \times G1 + C2 \times G2 \qquad (6)$$

where C1 + C2 = 1. Once a fit G3 is found, the relative contributions of C1 and C2 can be used to give the proportional contributions of absorptive and dispersive fits, and one can determine the relative angle between the magnetization orientations of the spin injector and detector.

The spin injection/detection efficiency *P* of the CoFeB/GaN NW interface is found from the follow expression[17,18]:

$$\Delta R_s\,(L) = \frac{P^2 \rho \lambda_s}{A} e^{-L/\lambda_s} \qquad (7)$$

where ΔR_S is the magnitude of the MR dip, ρ is the GaN NW resistivity, λ_S is the spin diffusion length, L is the centre-to-centre distance between injector and detector, and A is the cross-sectional area of the wire.

From the definition $\lambda_S = (D{\cdot}T_2)^{1/2}$ and Supplementary Eqs. 1, 3, 4, and 5, T_2 is the sole independent fitting parameter if the carrier diffusion constant D is known, for example from a measurement of ρ and the Einstein relation. As presented in Supplementary Note 3, the measured value of λ_S (300 K) is 710 $\pm$ 90 nm in a sample where T_2 was not measured. Measured values of T_2 varied from sample to sample (T = 300 K), in the range 100 to 150 ps. The variation of our deduced values λ_S, associated with varying values of T_2, is less than the variation range from experimental error, 620 to 800 nm. For the Hanle data sets, the measured distance between F1 and F2 is 1000 $\pm$ 100 nm. In the fitting process, we use L0 as an independent parameter and values of L0 are in the range 1.0 to 1.3. For any data set, the value of $L0$ is within experimental error for the fitted value of T_2. Examples of fitting parameters are shown in Supplementary Table 1, showing derived values of T_2, C1, C2, λ_S, and P. We note that our measured and deduced values of λ_S are much larger (by a numerical factor of 3 to 5) than values previously reported for GaN NWs: λ_S = 260 nm for GaN NW in the NLSV measurement at room temperature[19] and λ_S = 176 nm for bulk GaN in the three-terminal Hanle measurement at room temperature[20]. In addition, we observed large spin injection/detection polarization in the trigonal GaN NW of $P \approx 9\%$ at room temperature, that is even much larger than that of previously reported for GaN NWs: $P \approx 0.7\%$ for GaN

NW in a NLSV Hanle measurement at room temperature[19] and opens a way to develop spin injection device working at room temperature.

Supplementary Note 8. Hanle effect in a type-D device for fields at 30° and 150° relative to the *z*-axis.

A detailed analysis of the Hanle effect in type-C devices, for fields with angle 30°, 150°, and 90°, is presented in the text. A comparison is then made with a data set from a type-D device with field at 90° ($H = H_y$), where no Hanle effect is observed. In Supplementary Fig. 9, we present Hanle data from a type-D device for field angles 30° (Supplementary Fig. 9a) and 150° (Supplementary Fig. 9b). Raw data sets have been modified for Overhauser shifts (Supplementary Note 5) and then were fit to combinations of absorptive and dispersive Hanle shape functions (Supplementary Note 7). We note that the MR dips for the type-C device varied with field orientation and had magnitude in the range 9 to 16 Ω (300 K, Supplementary Table 1). For the type-D device (raw data not shown), the MR dips had magnitude 12 Ω independent of field orientation (300 K). We deduce that the magnitude of spin accumulation is comparable in both types of devices.

Comparison of the Hanle traces in Figs. 4a and 4b (type-C device) with those in Supplementary Figs. 9a and 9b (type-D device) shows several dramatic differences. When characterizing the magnitude of the Hanle curve as the resistance difference between maximum and minimum values, the type-C device shows a magnitude that varies strongly with field orientation, approximately 2 Ω for H at 30° and 7 Ω for H at 150°. The character of

the trace is purely absorptive (a dip, or negative absorption shape, signifying that injector and detector magnetization orientations are antiparallel) for H at 30° and a nearly equal mixture of positive absorptive and positive dispersive for H at 150°. By contrast, the type-D device shows a magnitude that is independent of field orientation, approximately 5 Ω for H at both angles. The shapes of the traces are quite similar to each other. For H at 30° the curve is a nearly equal mixture of positive absorptive and negative dispersive, and for H at 150° the curve has a small portion of positive absorptive and a dominant component of negative dispersive. These characteristics are consistent with our model that spin injection/detection is equal at both interfaces of the type-D device, the $(11\bar{2})$ and $(\bar{1}\bar{1}\bar{2})$ interfaces. The relatively small difference in the character of the traces can readily be explained by field dependent variations in the magnetization states of injector and detector.

Supplementary References

1. Johnson, M. & Silsbee, R. H. Thermodynamic analysis of interfacial transport and of the thermomagnetoelectric system. *Phys. Rev. B* **35,** 4959–4972 (1987).
2. Godfrey, R. & Johnson, M. Spin Injection in Mesoscopic Silver Wires: Experimental Test of Resistance Mismatch. *Phys. Rev. Lett.* **96,** 136601 (2006).
3. Takahashi, S. & Maekawa, S. Spin injection and detection in magnetic nanostructures. *Phys. Rev. B* **67,** 052409 (2003).
4. Garzon, S., Žutić, I. & Webb, R. A. Temperature-Dependent Asymmetry of the Nonlocal Spin-Injection Resistance: Evidence for Spin Nonconserving Interface Scattering. *Phys. Rev. Lett.* **94,** 176601 (2005).
5. Kimura, T., Hamrle, J. & Otani, Y. Estimation of spin-diffusion length from the magnitude of spin-current absorption: Multiterminal ferromagnetic/nonferromagnetic hybrid structures. *Phys. Rev. B* **72,** 014461 (2005).
6. Yan, W. *et al.* A two-dimensional spin field-effect switch. *Nat. Commun.* **7,** 13372 (2016).
7. Awo-Affouda, C. *et al.* Contributions to Hanle lineshapes in Fe/GaAs nonlocal spin valve transport. *Appl. Phys. Lett.* **94,** 102511 (2009).
8. Akiho, T. *et al.* Electrical injection of spin-polarized electrons and electrical detection of dynamic nuclear polarization using a Heusler alloy spin source. *Phys. Rev. B* **87,** 235205 (2013).
9. Salis, G., Fuhrer, A. & Alvarado, S. F. Signatures of dynamically polarized nuclear spins in all-electrical lateral spin transport devices. *Phys. Rev. B* **80,** 115332 (2009).
10. Chan, M. K. *et al.* Hyperfine interactions and spin transport in ferromagnet-

semiconductor heterostructures. *Phys. Rev. B* **80,** 161206 (2009).

11. Shiogai, J. *et al.* Dynamic nuclear spin polarization in an all-semiconductor spin injection device with (Ga,Mn)As/n-GaAs spin Esaki diode. *Appl. Phys. Lett.* **101,** 212402 (2012).

12. Christie, K. D. *et al.* Knight shift and nuclear spin relaxation in Fe/n-GaAs heterostructures. *Phys. Rev. B* **92,** 155204 (2015).

13. Harmon, N. J. *et al.* Anisotropic spin relaxation in n-GaAs from strong inhomogeneous hyperfine fields produced by the dynamical polarization of nuclei. *Phys. Rev. B* **92,** 140201 (2015).

14. Puttisong, Y. *et al.* Efficient room-temperature nuclear spin hyperpolarization of a defect atom in a semiconductor. *Nat. Commun.* **4,** 1751 (2013).

15. Porter, D. G. & Donahue, M. J. OOMMF user's guide, version 1.2. *Natl. Inst. Stand. Technol. Interag. Rep. NISTIR 6376.*

16. Johnson, M. & Silsbee, R. H. Coupling of electronic charge and spin at a ferromagnetic-paramagnetic metal interface. *Phys. Rev. B* **37,** 5312–5325 (1988).

17. Johnson, M. Spin polarization of gold films via transported (invited). *J. Appl. Phys.* **75,** 6714–6719 (1994).

18. Johnson, M. Spin injection and accumulation in mesoscopic metal wires. *J. Phys. Condens. Matter* **19,** 165215 (2007).

19. Kum, H. *et al.* Room temperature single GaN nanowire spin valves with FeCo/MgO tunnel contacts. *Appl. Phys. Lett.* **100,** 182407 (2012).

20. Jahangir, S., Doğan, F., Kum, H., Manchon, A. & Bhattacharya, P. Spin diffusion in bulk GaN measured with MnAs spin injector. *Phys. Rev. B* **86,** 035315 (2012).
